# A service evaluation of paediatric pain management in an English ambulance service

**DOI:** 10.29045/14784726.2019.09.4.2.37

**Published:** 2019-09-01

**Authors:** Richard Pilbery, Jamie Miles, Fiona Bell

**Affiliations:** Yorkshire Ambulance Service NHS Trust: ORCID iD: https://orcid.org/0000-0002-5797-9788; Yorkshire Ambulance Service NHS Trust: ORCID iD: https://orcid.org/0000-0002-1080-768X; Yorkshire Ambulance Service NHS Trust: ORCID iD: https://orcid.org/0000-0003-4503-1903

**Keywords:** analgesia, paediatric, pain

## Abstract

**Introduction:**

Evidence from the past 20 years has highlighted that acute pain is not managed well in the emergency setting, in particular with children. Inadequate management of pain can result in long-term changes in both physical and mental health. This service evaluation aimed to determine how paediatric pain is assessed and managed by ambulance clinicians in a large region in England.

**Methods:**

This retrospective service evaluation analysed electronic patient record (ePR) data routinely collected between September and December 2018. All paediatric patients (< 18 years of age) with pain documented narratively, or a pain score of ≥ 1/10, were included. The primary outcome measure was the proportion of patients with severe pain (defined as a pain score of ≥ 7/10) who achieve effective pain management (reduction in pain score of ≥ 2/10).

**Results:**

A total of 2801 paediatric patients who had documented pain were included in the analysis and the median age of patients was three years (interquartile range, 1–12 years). Most had a medical cause of pain (2387/2801, 85.2%), and analgesia was administered by the ambulance crew in 403/2801 (14.4%) patients. Multiple pain scores were recorded for 667 patients. Effective pain management was achieved in 233/271 (86%) patients in moderate pain and 204/210 (97.1%) patients in severe pain. However, of the 437 children in moderate to severe pain who achieved effective pain management, 381 (87%) received no analgesia.

**Conclusion:**

Children in severe pain received effective pain management, despite the majority not receiving any analgesia. This should be investigated further since non-pharmacological methods of analgesia are unlikely to explain a reduction of this magnitude. Ambulance staff need to be encouraged to record a pain score promptly after arriving on scene and ensure it is repeated. Pain score should be documented as part of the physiological observations and not in the free text of ePRs to ensure that it is identified during audits.

## Introduction

Relieving pain is an important role of healthcare professionals (Lohman, Schleifer, & Amon, 2010). However, there is evidence from the past 20 years that acute pain is not managed well in the emergency setting, and is particularly poorly managed in children (Murphy et al., 2016). Inadequate management of pain can result in long-term changes in stress hormone responses and pain reception, and increase the child’s risk of developing post-traumatic stress disorder (Finley, Franck, Grunau, & vonBaeyer, 2005; Saxe et al., 2001; Sheridan et al., 2014).

There are numerous studies relating to the out-of-hospital management of paediatric pain that suggest it is inadequate, whether due to inadequate pain assessment and/or analgesic administration (Browne et al., 2016; Hennes, Kim, & Pirrallo, 2005; Lerner et al., 2014; Lord, Jennings, & Smith, 2016; Samuel, Steiner, & Shavit, 2015; Swor, McEachin, Seguin, & Grall, 2005). There is limited evidence of the adequacy of paediatric pain management from the UK, with only one service evaluation and two clinical audits relating to the topic published. While the incidence of pain scoring from these studies was in excess of 90%, analgesic administration rates were between 52% and 84% (Shaw, Murphy-Jones, & Fothergill, 2018; Whitley & Bath-Hextall, 2017).

This service evaluation aimed to determine how paediatric pain is assessed and managed by Yorkshire Ambulance Service NHS Trust (YAS).

## Methods

A retrospective service evaluation analysing routine data collected between September and December 2018 was undertaken, to determine how paediatric pain is assessed and managed by YAS.

### Setting

YAS provides 24-hour emergency and healthcare services for the county of Yorkshire, in England. The county has a population of approximately 5 million, spread over almost 6000 square miles of varied terrain, including isolated moors and dales, coastline and urban areas. YAS operates 62 ambulance stations, and in 2017–2018 received 946,881 emergency calls which resulted in 778,639 attendances by YAS staff. Approximately 5% of these involved paediatric patients.

### Outcome measures

The primary outcome measure is the proportion of patients with severe pain (defined as a pain score of 7/10 or greater) who have a reduction in pain score of 2 or more.

Secondary outcomes include:

- proportion of paediatric patients with documented pain who have analgesia administered;
- proportion of paediatric patients where pain is described in the text of the ePR without a pain score being recorded;
- proportion of paediatric patients who have more than one pain score recorded;
- median time from arrival to pain scoring and analgesic administration;
- frequency of analgesic administration to paediatric patients stratified by pain severity and presumed cause of pain;
- frequency of specific pain score tool use in paediatric patients, e.g. Wong-Baker or Face, Legs, Activity, Cry, Consolability (FLACC) scales; and
- frequency of attendance of emergency medical technician (EMT) 2/paramedic in cases of severe paediatric pain.

### Data collection

All electronic patient records (ePRs) created between 1 September 2018 and 31 December 2018 which matched the following inclusion criteria were requested from the YAS business intelligence team:

- accessed the ambulance service via 999/111;
- received a face-to-face assessment with a clinician;
- less than 18 years of age at time of call; and
- had at least one pain score of 1/10 or greater recorded, or had documented pain recorded in the free text of the ePR.

Cases were excluded if the working impression code was: ‘no illness or injury’. The following data items were extracted from the ePRs:

- patient age;
- initial and subsequent pain scores and the time they were recorded;
- attending ambulance staff skill level, e.g. paramedic/EMT;
- administered medications and the time they were recorded;
- time spent with patient out-of-hospital (i.e. on scene time to arrival at hospital/clear from scene); and
- the following free text ePR fields:
  - presenting complaint;
  - history of complaint;
  - initial presentation;
  - working impression;
  - on examination;
  - diagram notes;
  - care plan;
  - transported;
  - non-transport reason; and
  - advice text.

### Statistical analysis

A descriptive analysis was conducted using custom computer code created within the statistics package, R (R Core Team, 2018). This code processed the free text fields in the ePR, identifying documented pain (with or without a pain score) and the use of paediatric pain scoring tools. Data were aggregated to provide summary statistics of analgesic use by presumed cause of the pain, pain severity and analgesia used. Patients were classified as either medical or trauma in origin, based on the working impression code in the first instance, or, in the case that several codes were considered to be equivocal in terms of their classification, a key word search for indications that the incident was trauma-related (Supplementary 1). Pain severity was stratified into severe (pain score ≥ 7/10), moderate (pain score 4–6/10) and mild (pain score 1–3/10).

## Results

Between 1 September 2018 and 31 December 2018, there were 9748 ePRs created for patients aged 0–17 years who had received a face-to-face assessment by YAS staff following a 999/111 call. Once 52 cases with a working impression of ‘no illness or injury’ were excluded, 2801 patients remained who had documented pain, either recorded in the free text of the ePR, or as a pain score of 1/10 or greater (Table 1).

**Table 1.** Summary details of paediatric pain management.

Variable	Trauma	Medical	Total
n (%)	414 (14.8)	2387 (85.2)	2801 (100)
Median age in years (IQR)	10 (3–14)	3 (1–10)	3 (1–12)
Female n (%)	143 (34.5)	1138 (47.7)	1281 (45.7)
Analgesia administered by crew n (%)	150 (36.2)	253 (10.6)	403 (14.4)
Analgesia before arrival n (%)	0 (0)	10 (0.4)	10 (0.4)
***Pain severity n (%)***			
Severe pain (≥ 7/10)	94 (22.7)	551 (23.1)	645 (23)
Moderate pain (4–6/10)	125 (30.2)	757 (31.7)	882 (31.5)
Mild pain (1–3/10)	73 (17.6)	617 (25.8)	690 (24.6)
Unknown	122 (29.5)	462 (19.4)	584 (20.8)
***Numerical pain scoring recorded n (%)***			
Single pain score	201 (48.6)	1349 (56.5)	1550 (55.3)
Multiple pain scores	91 (22)	576 (24.1)	667 (23.8)
Multiple pain scores for severe pain	31	179	210
No pain scores	109 (26.3)	366 (15.3)	475 (17)
Unable to record pain score	13 (3.1)	96 (4)	109 (3.9)
***Median time to pain score mins (IQR)***			
Severe pain	20 (10–36)	13.5 (6.5–27)	19 (7–31)
Moderate pain	18.5 (16–27)	12.5 (7–24)	16.5 (8–25.5)
Mild pain	30 (16–32.5)	11 (5.5–24.5)	11.5 (5–30)
***Analgesia administered (%)***			
Severe pain	36 (38.3)	41 (7.4)	77 (11.9)
Moderate pain	34 (27.2)	78 (10.3)	112 (12.7)
Mild pain	20 (27.4)	59 (9.6)	79 (11.4)
Unknown	60 (49.2)	75 (16.2)	135 (23.1)
***Median time to YAS-administered analgesia mins (IQR)***			
Severe pain	20 (15–26)	20 (17.5–31)	20 (16–26)
Moderate pain	26 (16–38)	14 (8–19)	19 (13–27.5)
Mild pain	26 (24–30.5)	18 (11–32.5)	22 (12–32)
***Paediatric pain scoring tools***			
Wong-Baker	1	3	4
FLACC	0	8	8
***Most senior grade of clinician on scene, n (%)***			
EMT or paramedic	333 (80.4)	1937 (81.1)	2270 (81.0)
Paramedic	278 (67.1)	1674 (70.1)	1952 (69.7)
EMT2	65 (15.7)	341 (14.3)	406 (14.5)
Lower grade of staff	81 (19.6)	450 (18.9)	531 (19.0)

Note: EMT = emergency medical technician; FLACC = Face, Legs, Activity, Cry, Consolability; IQR = interquartile range; YAS = Yorkshire Ambulance Service NHS Trust.

**Figure 1:**
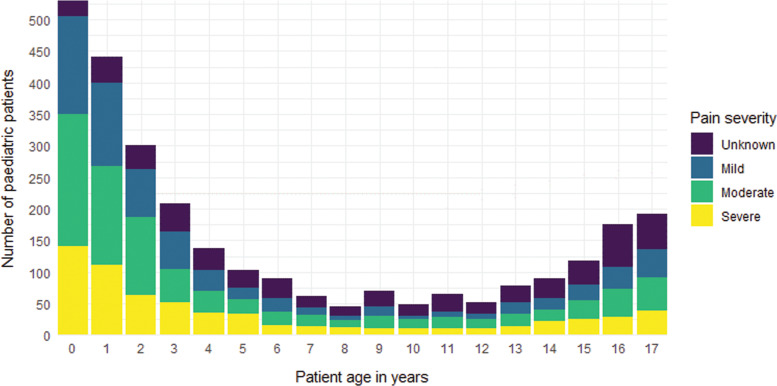
Figure 1. Frequency of paediatric patients in pain, stratified by age and pain severity.

Note: Pain severity defined as a pain score of ≥ 7/10 for severe pain, 4–6/10 for moderate pain and 1–3 for mild pain.

The distribution of paediatric patients with pain is bimodal, with the highest number of patients aged under 4 years and over 15 years of age (Figure 1).

At least one pain score was documented in 2217/2801 (79.2%) of paediatric patients, with 667/2801 (23.8%) having more than one pain score documented. There were 109/2801 (3.9%) cases where it was documented that a pain score could not be recorded, and there were 475/2801 (17%) cases where pain was documented in the free text but no pain score recorded. In total, 403/2801 (14.4%) patients in pain received analgesia from the crew, with 268/2217 (12.1%) patients receiving analgesia having had a pain score recorded. There were few documented cases of Wong-Baker/FLACC assessment tool use, with 12 documented cases of their use in the free text of the ePR. However, by default, for paediatric patients under the age of eight years, the Wong-Baker FACES^®^ scale is presented to the ambulance clinician to assess pain. In this dataset, 1915 (68.4%) paediatric patients were under the age of eight, and 1641 had at least one pain score recorded.

The median time from arrival to first pain score was 12 minutes (interquartile range (IQR), 6–25) and from arrival to analgesia administration, 19 minutes (IQR, 12–29). A paramedic or EMT was on scene in 2270/2801 (81%) of cases, with a paramedic on scene, and thus all analgesic options available, in 1952/2801 (69.7%) of cases.

### Analgesia administration

**Figure 2:**
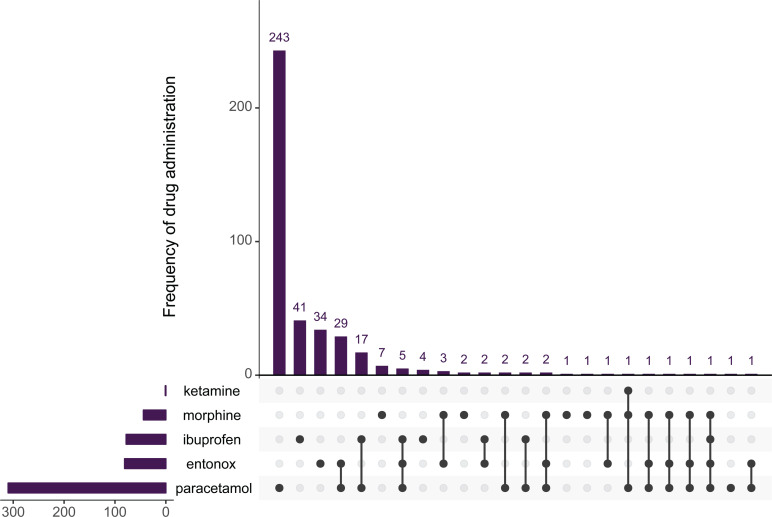
Figure 2. Frequency of drug administration, stratified by drug and drug combination.

Note: Horizontal bar chart shows total number of drug administrations, vertical bar chart shows number of drug administrations either alone or in combination.

The most commonly administered drug was paracetamol (n = 310), followed by entonox (n = 81) and ibuprofen (n = 78). The most common combination of analgesic drugs was paracetamol and entonox (n = 30), and paracetamol and ibuprofen (n = 19, Figure 2). A total of 71/547 (13.0%) of analgesic drugs were administered via an intra-vascular (i.e. intravenous or intraosseous) route. This was also the case for patients with severe pain, with 13/107 (12.1%) of cases receiving analgesia via an intra-vascular route (Table 2).

**Table 2.** Individual drug administrations stratified by drug and route.

	Route				
Drug	Inhaled	Intravenous	Oral	Unknown	Total
Entonox	81	0	0	0	81
Ibuprofen	0	0	78	0	78
Ketamine	0	1	0	0	1
Morphine	0	42	0	1	43
Paracetamol	0	16	292	2	310
Total	81	59	370	3	513

### Pain score reduction

For the subgroup of patients who had two pain scores recorded (n = 667), there was a pain score reduction of two points or more in 233/271 (86%) patients with moderate pain and 204/210 (97.1%) patients in severe pain. The majority of patients who received no analgesia also appeared to demonstrate high levels of pain score reduction (Table 3).

**Table 3.** Summary of analgesic effectiveness stratified by drug.

Variable	Entonox	Ibuprofen	Morphine	No. analgesia	Paracetamol	Polyanalgesia	All analgesic drugs	All patients
***Moderate pain***								
n	1	4	1	239	21	5	32	271
Pain score reduction by 2+ (n)	1	2	1	206	18	5	27	233
Median pain reduction (IQR)	4 (4–4)	3.5 (0.5–6)	4 (4–4)	4 (3.5–6)	4 (2–4)	4 (4–4)	4 (2–4)	4 (3–5.5)
***Severe pain***								
n	2	3	2	180	20	3	30	210
Pain score reduction by 2+ (n)	2	2	2	175	20	3	29	204
Median pain reduction (IQR)	9.5 (9–10)	4 (2.5–5.5)	9.5 (9–10)	8 (5.5–9)	8 (7–9.5)	2 (2–5)	8 (7–9)	8 (6–9)

Note: IQR = interquartile range.

### Patients with no pain score

Of the 2801 paediatric patients with pain reported, 475 (17%) did not have a numerical pain score recorded as part of the physiological observation section of the ePR. However, 81/475 (17%) records contained at least one pain score in the free text portion of the ePR, which would not typically be picked up in an audit. If these pain scores are added to the total number of patients who have at least one pain score (or a documented reason why one could not be obtained), this would total 2407/2801 (85.9%). However, there were 105 drug administrations with no pain score recorded, but pain documented in the free text of the record (Table 4).

**Table 4.** Drug administrations with no pain score recorded.

Drug	No pain score (free text or observations)	No pain score in observations
Paracetamol	61	78
Polyanalgesia	24	31
Entonox	14	18
Ibuprofen	4	5
Morphine	2	3
Total	105	135

### Medical working impressions

Since a definitive diagnosis is not always possible in the out-of-hospital environment, ambulance staff typically record a single working impression from a pre-defined list. While not clearly defined, a working impression could be considered to be the clinician’s current hypothesis as to the likely cause or immediate healthcare need that explains the patient’s presentation, from which the management plan is based. The top 20 working impressions for patients with recorded pain of medical impression can be seen in Table 5.

**Table 5.** Top 20 most common medical working impressions.

Description	n	%
Generally unwell	624	26.1
Shortness of breath	337	14.1
Other medical condition	164	6.9
Febrile illness	141	5.9
Abdominal pain	136	5.7
Pyrexia of unknown origin	81	3.4
Epileptic fit	67	2.8
Pain: other	66	2.8
Seizures (non-epileptic)	62	2.6
Cold and flu	60	2.5
Allergic reaction/rash	58	2.4
Vomiting	56	2.3
Convulsions/fitting	54	2.3
Asthma	51	2.1
Chest infection: pneumonia	43	1.8
Drug overdose	43	1.8
Collapse: reason unknown	40	1.7
Psychiatric problems	32	1.3
Alcohol related	29	1.2
Choking	24	1.0
Sepsis	24	1.0
Other impressions	195	8.2
Total	2387	100.0

### Trauma working impressions

There were 414 paediatric patients with a traumatic cause to their pain. Table 6 highlights the top 10 working impressions for trauma.

**Table 6.** Top 10 most common trauma working impressions.

Description	n	%
Head injury	105	25.4
Minor injuries: other	59	14.3
Falls	58	14.0
Fracture/possible fracture	43	10.4
Pain: other	38	9.2
Minor cuts and bruising	29	7.0
Haemorrhage/lacerations	13	3.1
Sprain/strain/dislocation	13	3.1
Burns	12	2.9
Major trauma	9	2.2
Other impressions	35	8.5
Total	414	100.0

## Discussion

In this evaluation of 2801 paediatric patients who had an ePR completed by a YAS clinician between 1 September 2018 and 31 December 2018 and had documented pain, 403/2801 (14.4%) had analgesia administered by ambulance staff and 2217/2801 (79.2%) had at least one pain score recorded. If the ‘unable to record a pain score’ (UTR) fields and documented pain scores in the free text are included, this total rises to 2407/2801 (85.9%).

### Pain scoring

Making direct comparisons with other literature is difficult, due to varying inclusion criteria for patient age and presenting complaint. One of two published studies conducted in UK NHS ambulance services is a service evaluation conducted by Whitley and Bath-Hextall (2017). They included 11,317 children between the ages of 1 and 17 years who had pain secondary to trauma. A total of 90.8% of patients had a documented pain score or UTR reason, which is higher than reported in this evaluation. The only other UK ambulance study is an abstract relating to a clinical audit conducted by the London Ambulance Service (Shaw et al., 2018). They conducted a retrospective review of 229 patients under the age of 12 years with suspected fracture or dislocation, and found that 97% had a documented pain score. However, the limited data presented in the abstract do not enable a detailed comparison between this evaluation and the audit. A recent study investigating factors associated with pain treatment and outcomes in adults showed that in 15.4%–41.2% of analgesia administrations pain scores were missing from the patient record, dependent on the drug administered (Siriwardena et al., 2019), indicating barriers to pain score recording by pre-hospital clinicians more widely.

In Ireland, Murphy et al. (2016) conducted a 12-month prospective cross-sectional study to examine the pre-hospital and emergency department (ED) management of paediatric pain. A total of 6371 children with pain as a documented symptom and who attended one of the four participating Irish EDs, having been transported by ambulance, were included. As with this evaluation, they noticed a bimodal distribution of patient age, with peaks around the toddler and adolescent age groups. Pain assessment rates were low, with only 32% of patients receiving a pain assessment by the ambulance service.

Further afield, Lord et al. (2016) published a retrospective cohort study of 38,167 patients under the age of 15 years in the state of Victoria, Australia. The median patient age was higher than this evaluation (10 years of age, IQR 5–12) and the definition of severe pain was different (8/10 or more, vs. 7/10 or more used in this evaluation). Pain score recording was similar to this evaluation, with 81.2% of patients having a pain score recorded.

In this study, the median time from arrival on scene to initial pain score was 19 minutes (IQR 7–31 minutes) for severe pain, which was longer than that for mild and moderate pain. It is not clear why this should be the case, although perhaps it reflects the severity of injury and illness, and other treatment took priority initially. This view might be substantiated by the higher median time in the trauma group. However, once a pain score had been recorded, time to analgesia was prompt in cases of severe pain.

### Pain scoring tools

There were 105 cases where a pain score was not recorded and it was not possible to determine the reasons for the absence of a score. Difficulty in obtaining pain scores in children has been described in other studies (Browne et al., 2016; Hennes et al., 2005; Lerner et al., 2014; Lord et al., 2016; Samuel et al., 2015; Swor et al., 2005), and it is also possible that evidence of poor assessment of pain is reflected by the high numbers of patients with severe and moderate pain who had dramatic falls in their pain score despite no analgesia being administered (Table 2).

While the Wong-Baker FACES^®^ pain rating scale and FLACC pain assessment tool are in widespread use, they have not been validated for use out-of-hospital by ambulance clinicians. It may not be appropriate to consider scores calculated by FACES^®^ as equivalent to the numerical rating scale (NRS), except in older children (Garra et al., 2010; Oliveira, Batalha, Fernandes, Gonçalves, & Viegas, 2014). In addition, possible FACES^®^ scores increase in increments of two, not one (i.e. 0, 2, 4, 6, 8, 10).

While FLACC is allocated a score from 0 to 10 in single increments, in the same way as the NRS, it is not clear whether automatically transposing a FLACC score to an NRS is appropriate. Studies validating the FLACC score are inconclusive as to its ability to discriminate pain in younger children. For example, while Manworren and Hynan (2003) did find FLACC could be successfully utilised to assess pain in pre-verbal children, Willis, Merkel, Voepel-Lewis and Malviya (2003) found no correlation between FACES^®^ and FLACC scores in younger children.

There were few documented cases of specified FLACC or Wong-Baker pain scale use in this evaluation, however since it is the default pain scoring option for all patients under the age of eight, it is likely that a high proportion of patients did have their pain scored using this scale.

### Analgesia administration

This study found a low rate of analgesia administration (14.4%) despite a high amount of pain scoring (85.9%). This is a significant finding compared to the literature. Whitley and Bath-Hextall (2017) undertook a large service evaluation into paediatric analgesia in trauma. They found that analgesia was administered in 51.6% of cases. In this study, trauma patients received analgesia in 36.2% of cases, which is still lower than Whitley and Bath-Hextall results. So far, there is a sparsity of qualitative literature to understand why analgesia administration is low. Shaw et al. (2018) evaluated whether the introduction of an educational intervention training staff in paediatric pain management improved this. They found that since the intervention there was a 61% increase in analgesia being administered to children. This could indicate that confidence could be a factor. Lord et al. (2016) conducted a large epidemiological study of pain management in Australia. They initially reported low rates of analgesia administration (39.5% overall) but stated that since the introduction of intranasal fentanyl the odds of a patient receiving analgesia increased significantly. This supports the concept of confidence. In addition their study found that strong analgesics delivered non-intra-vascularly were preferred by clinicians (methoxyflurane 34.1%, fentanyl 8.6%, morphine 3.4%).

The most commonly administered analgesic drugs in this evaluation were paracetamol, entonox and ibuprofen, which is similar to that reported by Murphy et al. (2016) and Whitley and Bath-Hextall (2017). An inhaled drug (methoxyflurane) was the most common choice reported by two Australian papers (Jennings, Lord, & Smith, 2015; Lord et al., 2016). Both studies also included intranasal fentanyl which was a new drug and route introduced during their data collection period. They found fentanyl was also preferable to intravenous morphine, suggesting that routes not requiring vascular access were favoured by ambulance crews. There was evidence of this preference in the evaluation, as even for patients with severe pain, only 13/107 (12.1%) received analgesia via an intra-vascular route, despite many of the patients being too young to use entonox.

However, non-pharmacological methods of pain relief, such as distraction, or cooling burns, were not extracted from the ePRs, but it is unlikely that such alternative methods of pain relief completely explain the reductions in pain score seen for the large number of patients that had no analgesia being administered.

### Limitations

This evaluation is a retrospective review of routine electronic records, which represents approximately 68% of all paediatric incidents that occurred during the time of the evaluation. The low number of patients with multiple pain scores, particularly those in severe pain, makes it difficult to generalise. It also relies on clinicians accurately recording what they did and when, including documenting when drug administration was inappropriate. This is particularly relevant for infants, since analgesic options for paramedics are limited. In addition, over 50% of paediatric patients in this evaluation were under the age of three years, and are in a difficult group to assess and administer analgesia to.

Due to the number of ePRs, it was necessary to automatically process the free text using a bespoke algorithm. However, it is possible that this method included or excluded patients inappropriately. On the positive side, it did enable identification of paediatric patients who did not have pain scores identified, a group that has been missed by studies that have a pain score of 1/10 or greater as an inclusion criterion.

## Conclusion

Pain assessment in this evaluation is consistent with figures published elsewhere, although the frequency of analgesic administration appears to be significantly lower, especially in medical patients. Children in severe pain received effective pain management, despite the majority not receiving any analgesia. This should be investigated further since non-pharmacological methods of analgesia are unlikely to explain a reduction of this magnitude. Ambulance staff need to be encouraged to record a pain score promptly after arriving on scene and ensure it is repeated. Pain scores should be documented as part of the physiological observations and not in the free text of ePRs to ensure that they are identified during audits.

## Conflict of interest

None declared.

## Ethics

Not required.

## Funding

None.
